# Pro-Coagulant Endothelial Dysfunction Results from EHEC Shiga Toxins and Host Damage-Associated Molecular Patterns

**DOI:** 10.3389/fimmu.2015.00155

**Published:** 2015-04-07

**Authors:** Chad L. Mayer, Caitlin S. L. Parello, Benjamin C. Lee, Kiyoshi Itagaki, Shinichiro Kurosawa, Deborah J. Stearns-Kurosawa

**Affiliations:** ^1^Department of Pathology and Laboratory Medicine, Boston University School of Medicine, Boston, MA, USA; ^2^Department of Surgery, Beth Israel Deaconess Medical Center, Harvard Medical School, Boston, MA, USA

**Keywords:** hemolytic uremic syndrome, thrombotic microangiopathy, Shiga toxin 1, Shiga toxin 2, protein C, damage-associated molecular patterns, endothelial cells

## Abstract

Hemolytic uremic syndrome (HUS) from enterohemorrhagic *Escherichia coli* infection is a leading cause of kidney failure in otherwise healthy U.S. children. The bacterial Shiga toxins (Stx) induce the characteristic coagulopathy of HUS, but the damage to toxin-receptor expressing cells and organ injury due to ischemia likely also releases inflammatory damage-associated molecular patterns (DAMPs), which may exacerbate injury along with the toxins. To examine this, human aortic and renal glomerular cell anti-coagulant and barrier functions were studied after *in vitro* challenge with Stx1, Stx2, and DAMPs. There was significant loss of surface anti-coagulant protein C pathway molecules, increased expression of pro-thrombotic PAR1 and reduced protein C activation capability by 15–27%. Histones nearly completely prevented the activated protein C protection of endothelial cells from thrombin-induced permeability. In mice, lethal Stx2 challenge elevated plasma HMGB1 (day 2, 321 ± 118%; *p* < 0.01) and extracellular histones (day 3, 158 ± 62%; *p* < 0.01). Mice colonized with Stx2-expressing *Citrobacter rodentium* developed increased HMGB1 (day 5, 155 ± 55%; *p* < 0.01) and histones (day 3, 378 ± 188%; *p* < 0.01). Anti-histone antibody reduced both DAMPs to baseline, but was not sufficient to improve survival outcome or kidney function. Together, these data suggest a potential role Stx to produce DAMPs, and DAMPs to produce endothelial injury and a pro-thrombotic environment.

## Introduction

Enterohemorrhagic *Escherichia coli* (EHEC) are toxigenic intestinal bacteria that cause vomiting, diarrhea, edema, and hemorrhagic colitis. In some patients, the disease can progress to a potentially life-threatening syndrome known as diarrhea-associated hemolytic uremic syndrome (D + HUS), characterized by thrombotic microangiopathy, thrombocytopenia, and hemolytic anemia, all of which contribute to acute kidney injury (1). In the U.S., D + HUS is a leading cause of acute kidney failure in otherwise healthy children (2). EHEC produce and secrete Shiga toxin 1 (Stx1), Shiga toxin 2 (Stx2), or both, and serotypes that secrete Stx2 are associated with more clinically severe disease (3). Much of the pathogenesis observed during EHEC infection is attributed to the toxins, which are considered primary virulence factors of EHEC. The toxins bind to globotriaosylceramide (Gb_3_, CD77) receptors whose distribution is particularly high on renal glomerular endothelial cells in humans and on renal tubular epithelium in mice (4–6). The toxins are then internalized and transported to the endoplasmic reticulum, and the A subunit is activated to generate RNA *N*-glycosidase activity (7). Within the cytosol, the active A subunit cleaves an adenine residue from 28S ribosomal RNA, which prevents protein synthesis (8–10) and initiates ribotoxic (11, 12) and endoplasmic reticular-stress responses (13), leading to apoptosis and inflammation.

A major clinical feature of D + HUS is thrombotic microangiopathy, in which clots form inappropriately in the microvasculature, resulting in ischemic consequences and organ injury. As endothelial cells are crucial for maintaining blood fluidity and preventing leakage through the vessel walls, it is possible that Stx- and inflammation-induced perturbations in these functions contribute to the observed thrombotic microangiopathy. A critical pathway mediating these functions is the protein C pathway, which provides anti-coagulant, anti-inflammatory, and barrier protection activities (14). The activated form of the protein C zymogen mediates these activities, and activated protein C (APC) is generated by the protease action of thrombin, which is localized to the endothelial cell surface through binding to thrombomodulin (TM). Generation of APC is further facilitated by the endothelial protein C receptor (EPCR), which presents protein C zymogen to the cell surface thrombin/TM complex for more efficient activation (15, 16). During *in vitro* studies using human renal glomerular endothelial cells (HRGEC), Stx2-induced a small decrease in TM antigen expression (17), but resultant functional changes were not determined.

As an anti-coagulant, APC inhibits coagulation cofactors Va and VIIIa (18), but its barrier-protective activity is mediated by its occupation of EPCR and subsequent activation of protease-activated receptor 1 (PAR1). PAR1 is intimately involved in endothelial barrier function, and signaling by this discriminatory receptor is protease-specific depending on whether it is activated by APC, thrombin, or other proteases (19–22). PAR1 activation by thrombin contributes to thrombosis while also increasing endothelial barrier permeability; however, PAR1 activation by APC in concert with EPCR elicits an opposite barrier-protective effect. Although human endothelial cells express the Gb_3_ toxin receptor, little is known about how the Shiga toxins impact expression and function of PAR1, EPCR, and TM, and disruption of these molecules can have significant consequences (23, 24).

Enterohemorrhagic *Escherichia coli* are generally non-invasive, but the intestinal damage observed during EHEC infection can be considerable, with inflammation, hemorrhage, edema, and focal necrosis predominating (25). Often released by damaged cells are molecules termed damage-associated molecular patterns (DAMPs) (26): normal, endogenous molecules that can be extruded from the cell into the blood or tissue. Examples of DAMPs include histones, which can circulate or localize in neutrophil extracellular traps (27), or HMGB1 from monocytes (28). DAMPs also are released from necrotic cells, and circulating DAMPs can activate many of the same receptors as pathogen-associated molecular patterns to propagate inflammation and tissue damage (29, 30). Some DAMPs also can cause endothelial dysfunction manifested by increased permeability (31) or increased platelet adhesion (27). Although it has not been repeatedly demonstrated that DAMPs are released in the context of EHEC infection or Shiga toxin release, DAMPs from damaged tissue increase in several patient and animal models of sepsis and trauma (32–35). Given the extent of intestinal and kidney injury after EHEC toxin challenge (36–38), and the relative paucity of Shiga toxins observed in serum during hemolytic uremic syndrome (HUS) (39), we hypothesized that injury to any cell expressing the Gb3 receptor for Shiga toxins would release DAMPs, and that those DAMPs compromise the antithrombotic and barrier-protective properties of endothelial cells, leading to thrombotic microangiopathy and HUS.

## Materials and Methods

### Reagents

Plasma levels of HMGB1 and extracellular histones were measured using ELISAs for HMGB1 (IBL-international, Toronto, ON, Canada, and Chondrex Inc., Redmond, WA, USA) and cell-death detection (Roche, Indianapolis, IN, USA), respectively. Human aortic endothelial cells (Cascade Biologics, Grand Island, NY, USA) or HRGEC (Sciencell, Carlsbad, CA, USA) were purchased and grown in endothelial cell medium (Sciencell) supplemented with 5% fetal bovine serum, 100 U/mL penicillin, 100 μg/mL streptomycin, endothelial cell growth supplements according to the manufacturer’s instructions. These cell lines are morphologically similar, but HAEC grow more quickly and form tighter junctions, in addition to not being fenestrated as renal glomerular endothelial cells are. All experiments were performed between passage 2 and 6. Antibodies were purchased against PAR1 (ATAP2, Santa Cruz Biotechnology, Dallas, TX, USA, un/conjugated to Alexa 488), and TM, clone 1029, conjugated to Oregon green, for flow cytometry, and clone 1A4 for on-cell western (Becton Dickinson, Franklin Lakes, NJ, USA). Anti-EPCR (JRK 1494, un/conjugated to Alexa 488) were available from C.T. Esmon (Oklahoma Medical Research Foundation, Oklahoma City, OK, USA). Goat anti-mouse IRDye 800CW for use in on-cell westerns was purchased from Li-COR (Lincoln, NE, USA). Stx1 and Stx2 were purchased from Tufts University and endotoxin was removed by incubation with polymyxin B-agarose (Sigma Aldrich, St. Louis, MO, USA). Residual endotoxin levels were <0.015 ng/mL (LAL Chromogenic Endotoxin Quantitation Kit, Thermo Scientific, Rockford, IL, USA). Hybridomas producing anti-histone IgG clone BWA3 (40) were kindly provided by Dr. Ann Rothstein (Department of Immunology and Virology, University of Massachusetts Medical School, Worcester, MA, USA) and the antibody was purified from conditioned media using HiTrap Protein G columns (GE Healthcare, Piscataway, NJ, USA) and standard methods. Calf thymus histones and TNFα were purchased from Sigma Aldrich. Human plasma-derived protein C was a kind gift from Kaketsuken (Kumamoto, Japan). Spectrozyme PCa was purchased from American Diagnostica (Lexington, MA, USA), human α-thrombin, and anti-thrombin III were from Hematologic Technologies, Inc. (Essex Junction, VT, USA). All other reagents such as bovine serum albumin (BSA), ethlyenediaminetetraacetic acid (EDTA), and 4-(2-hydroxyethyl)-1-piperazineethanesulfonic acid (HEPES) were laboratory or research grade and purchased from Sigma Aldrich and ThermoFisher Scientific.

### Mouse model of Stx2 toxemia

Mice were purchased from the Jackson Laboratories (Bar Harbor, ME, USA) for all experiments. All animal studies were approved by the Boston University Institutional Animal Care and Use Committee. Mice (6-week-old male C57BL/6J) were injected intraperitoneally with 1 ~ 1.2 ng Stx2/20 g bodyweight at day 0 and day 3. Control mice received equal volumes of normal saline by intra-peritoneal injection. Blood was collected into 5 mM EDTA by facial vein bleed prior to each Stx2 challenge, and periodically until euthanasia for downstream analyses, consistent with the IACUC-approved protocols. Animals were monitored and weighed daily and plasma blood urea nitrogen (BUN) was quantified as a marker of acute kidney injury using a QuantiChrome Urea Assay Kit (BioAssay Systems, Hayward, CA, USA).

### Mouse model of EHEC infection

Mice (6-week-old female C57BL/6J) were inoculated with *Citrobacter rodentium*. This is a Gram negative mouse pathogen that either was lysogenized with an Stx2-producing phage (strain DBS770) in order to express the Shiga toxin, or a control strain that does not express the toxin (strain DBS771). *C. rodentium* strains were kindly provided by Dr. John M. Leong (Department of Molecular Biology and Microbiology, Tufts University Medical Center, Boston, MA, USA) and have been described in detail elsewhere (41). Briefly, bacteria were grown in LB broth containing chloramphenicol (final 10 μg/mL) or both chloramphenicol and kanamycin (25 μg/mL) to an OD_600_ of 0.75–0.90. Bacteria were washed, re-suspended in sterile saline, and mice were challenged with 1 × 10^9^ CFU by oral gavage on day 0. Weight was monitored daily and plasma collected periodically by facial vein bleed. CFU/g feces was determined by mixing feces with 10 volumes of phosphate buffered saline (PBS) (ThermoScientific), homogenizing with a sterile toothpick, centrifuging 30 s at 4000 rpm, and plating serially diluted supernatants on LB agar with appropriate restrictive antibiotics. Plates were grown overnight at 32°C and colonies were counted the next day.

### Flow cytometry

Human aortic endothelial cells between passage 2 and 6 were seeded on 0.1% gelatin-coated plates at 1.5 × 10^4^ cells/cm^2^. The next day, media was replaced with fresh media containing Stx1 or Stx2 (100 ng/mL), extracellular histones (50 μg/mL), or combinations. Cells maintained ≥85% viability under these conditions. Cells were incubated for 24 h, washed with PBS and then detached using Versene (Invitrogen) and passed through a 40 μm nylon mesh. All subsequent steps were done on ice. Cells were washed in PBS with 25 mm HEPES, 0.5% BSA, and 5 mM EDTA, pH 7.4 (flow buffer) before staining with primary conjugated antibodies (2 μg/mL) for 30 min. Cells were washed again and re-suspended in flow buffer. Dead cells were excluded by propidium iodide staining. Cell associated fluorescence was determined using a FACSCalibur flow cytometer (Becton Dickinson) and data analyzed using FlowJo X (Treestar Inc., Ashland, OR, USA) software. Based on peak morphology (one peak vs. bimodal), the geometric mean or an M1 region expressed as a percentage was used for data analysis. One-way ANOVA with a Dunnett posttest was used to determine significant differences between geometric means or M1 regions at *p* < 0.05.

### On-cell western

Antigen expression on HRGEC was done by on-cell western due to difficulty in producing large numbers through passaging. On-cell western was performed with aortic endothelial cells as well, specifically to compare surface antigen expression with HRGEC at baseline. Optimal toxin and histone concentrations that impacted surface phenotype while maintaining cell viability were determined in preliminary dose–response studies. Cell viability was maintained at 82–96% under experimental conditions (data not shown). Black-walled, clear-bottomed 96-well tissue culture plates (Corning, Tewksbury, MA, USA), of the type used for fluorescent experiments, were coated with 0.1% gelatin and then seeded with HRGEC (Sciencell) grown in endothelial cell media. When cells were near confluent, media were replaced and challenges were added. After 24 h, cells were washed once with PBS then fixed for 20 min at room temperature in PBS with 3.7% formaldehyde. After fixation, cells were washed twice with 50 mM Tris, pH 7.4, 150 mM NaCl, 0.05% Tween-20 (TBST), and blocked with Odyssey blocking buffer (Li-Cor, Lincoln, NE, USA) at room temperature with gentle shaking. Following the blocking step, cells were washed twice with TBST and incubated with primary unconjugated antibodies for EPCR (JRK 1494), PAR1 (ATAP2), or TM (1A4) at 2 μg/mL in Odyssey blocking buffer. Cells were washed twice with TBST and incubated with goat anti-mouse IRDye 800CW (Li-Cor) diluted 1:800 in Odyssey blocking buffer and Draq5 (5 mM stock, Thermo Scientific) diluted 1:10,000. Plates were washed with TBST, and all liquid aspirated before reading fluorescent values on a Li-Cor Odyssey dual-channel infrared imager at 700 and 800 nm. Images were analyzed with Odyssey 2.1 software (LiCor), and fluorescent intensities at 800 nm were normalized to intact cell number based on Draq5 staining at 700 nm. One-way ANOVA with a Dunnett posttest was used to determine significance.

### Protein C activation assay

Confluent human aortic endothelial cells (HAEC) were incubated with Stx1 (100 ng/mL), Stx2 (100 ng/mL), calf thymus histones (50 μg/mL), or combinations. Confluent HRGEC were challenged with Stx1 (50 ng/mL), Stx2 (50 ng/mL), or histones (50 μg/mL). For experiments with anti-histone BWA3 monoclonal antibody, endothelial cells were challenged with 30 μg/mL histones with or without BWA3 at 20 μg/mL. All challenges were done for 24 h at 37°C in 5% CO_2_. Cells were washed with Hank’s balanced salt solution (HBSS; Thermo) and protein C was added at a final concentration of 0.4 μM in 0.1 mM HEPES, pH 7.4, 0.6 mM MgCl_2_, and 1 mM CaCl_2_. Human α-thrombin (10 nM final) was added and the reaction was stopped after 30 min at 37°C with HBSS, 0.1 mM HEPES, 0.5 mg/mL human ATIII, and 1.4 U/mL heparin. Spectrozyme PCa (American Diagnostica, Lexington, MA) was added (0.2 μM final) and the change in absorbance 405 nm with time was performed on a VersaMax ELISA Microplate Reader and analyzed with SoftMax Pro 5.4 (Molecular Devices, Sunnyvale, CA, USA). APC levels were determined by reference to a standard curve prepared with purified human APC, and normalized to intact cell numbers determined using Draq5 staining of a parallel plate, then normalized to 100% activation by unchallenged cells. One-way ANOVA with a Dunnett posttest was used to determine significant differences.

### Endothelial cell permeability

Endothelial cell monolayer permeability changes were quantified from changes in electrical resistance across a human aortic endothelial monolayer as monitored by electric cell-substrate impedance sensing using an ECIS^®^ ZO array station (Applied Biophysics, Troy, NY, USA). For each experiment, two 40 electrode/well 8W10E+ electrode chamber slides (Applied Biophysics) were coated with 10 mM l-cysteine/well for 10 min, washed 2 times with HBSS, coated with 5 μM fibronectin in HBSS for 10 min, then washed 2 times more with HBSS and allowed to dry. HAEC between passages 2 and 6 were seeded at a density of 2.5 × 10^5^ cells/chamber and allowed to adhere and form a monolayer as determined by the stabilizing capacitance at 64 kHz. For experiments with pre-treatments, histones (25 μg/mL) or Stx2 (100 ng/mL) were added to the appropriate wells 12 h before thrombin addition. Thrombin was added to 1 U/mL (16.85 nM) and the experiment was followed for at least 12 h after thrombin addition. For experiments using APC, concentrations of thrombin were lowered to 2 nM in order for the protective effect of APC on PAR1 to be observed (19). Pre-incubation with APC (10 nM) was done for 3 h prior to thrombin challenge. The area under the curve for normalized resistance over the selected range of time was determined and one-way ANOVA with a Dunnett posttest was used to determine significance.

## Results

### Large vessel endothelium expresses more surface EPCR, PAR1, and TM antigen than microvascular renal endothelium

**Figure 1:**
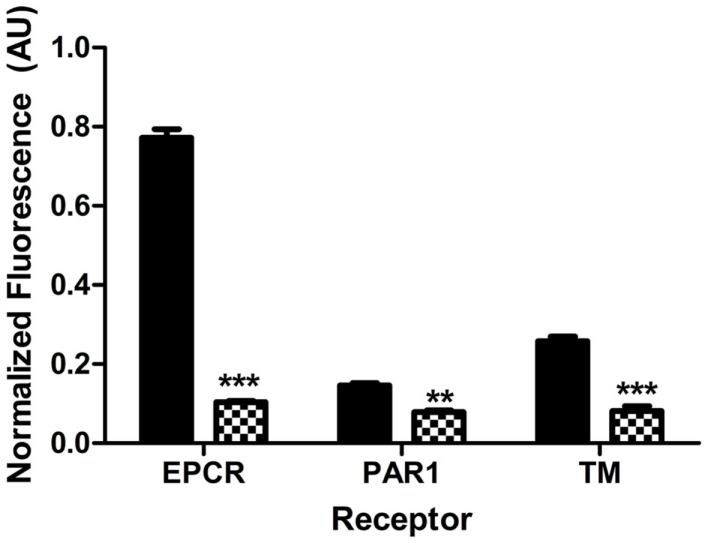
**Different endothelium express different amounts of PAR1, EPCR, and TM**. Surface expression of EPCR, PAR1, and TM on large vessel human aortic endothelial cells (HAEC, black) and microvascular human renal glomerular endothelial cells (HRGEC, checkered). The significance is determined by comparing the amount of EPCR, PAR1, or TM on each cell type in unchallenged cells at 24 h. Constitutive expression of EPCR, PAR1, and TM on both HAEC and HRGEC for this figure was determined by on-cell western, as described in Section “Materials and Methods.” Comparison was done by two-way ANOVA with a Bonferroni posttest. **p < 0.01, ***p < 0.001.

Differential endothelial expression of EPCR and TM is well-recognized, and while TM is widely expressed, surface EPCR expression is known to increase with vessel size (42). Relative quantitation of TM and EPCR levels in different vascular beds is not well established, particularly for renal endothelium. In order to better interpret the impact of Stx and DAMPs on receptor expression, quantitative comparisons of baseline surface EPCR, PAR1, and TM on the surface of normal, unchallenged HAEC, representing large vessel endothelium, and HRGEC, representing small vessel endothelium, were performed. These experiments differed from those involving Stx and DAMP challenges in that they were accomplished by performing on-cell westerns with both cell types. We observed that at baseline HAEC expressed significantly higher antigen amounts of EPCR (7.4-fold), PAR1 (1.8-fold), and TM (3.2-fold) when compared to HRGEC (Figure 1).

### Stx and DAMPs shift endothelial expression of PAR1 and protein C pathway molecules

**Figure 2:**
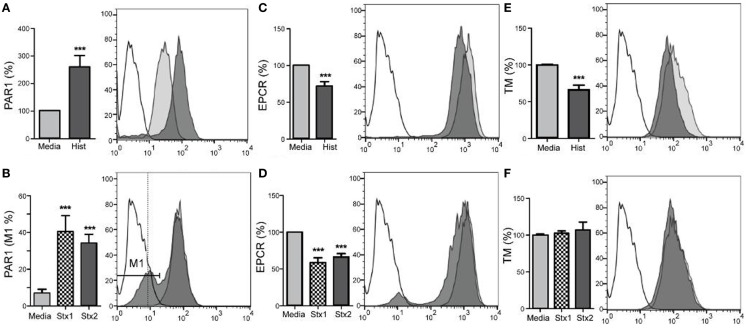
**Stx1, Stx2, and histones induce changes in aortic endothelial expression of surface molecules**. Human aortic endothelial cells were challenged with 50 μg/mL histones **(A,C,E)** or 100 ng/mL Stx1 **(B,D,F)** and surface expression of PAR-1, EPCR, and TM were quantified by flow cytometry using antigen-specific antibodies. Histogram overlays show representative staining of unchallenged cells (gray) and challenged cells (dark) compared to background staining (unfilled), with challenges represented using Stx1, as there were no differences in effect with respect to which Shiga toxin was used. Bar graphs show geometric means ± SD of three to four experiments each, except **(B)**, which compares the M1 region 1, expressed as a percentage. Differences from constitutive expression (media only = 100%) are shown as ***p < 0.001.

The abilities of the Shiga toxins and histone DAMPs to alter surface expression of endothelial cell molecules have important implications for maintaining blood fluidity and were determined by flow cytometry with HAEC (Figure 2). HAEC challenged with histones demonstrated significantly increased expression of thrombin receptor PAR1 relative to control cells (Figure 2A; *p* < 0.001), whereas challenge with either Stx1 or Stx2 caused a subset of cells (35–40%) to significantly lower their surface expression of PAR-1 (Figure 2B; *p* < 0.001). EPCR surface antigen expression was reduced significantly relative to media alone in cells incubated with histones or Stx (Figures 2C,D; *p* < 0.001). TM expression was reduced after histone treatment (Figure 2E; *p* < 0.001); however, Shiga toxins did not affect TM levels (Figure 2F).

**Figure 3:**
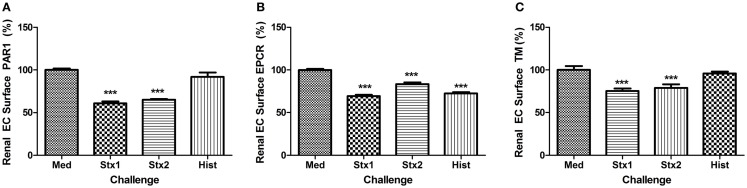
**Stx1, Stx2, and histones induce changes in renal glomerular endothelial expression of surface molecules**. Surface expression of **(A)** PAR1, **(B)** EPCR, and **(C)** TM were quantified by on-cell western after 24 h incubation with Stx1 (50 ng/mL), Stx2 (100 ng/L), or 50 μg/mL histones. Antigen data were normalized to cell number using Draq5 staining. Differences from media alone are shown as ***p < 0.001.

As the primary target organ of the toxins in humans is the kidney glomerular endothelium, HRGEC were challenged similarly to aortic endothelial cells (Figure 3). The renal cells are more sensitive to the histones and toxins *in vitro* (data not shown), so challenge concentrations were decreased to maintain >80% viability and on-cell western was used to minimize manipulation of the cells. Stx1 and Stx2 challenge reduced PAR1 (Figure 3A), EPCR (Figure 3B), and TM surface expression (Figure 3C). Histones did not appreciably alter PAR1 or TM expression, but did decrease EPCR expression on HRGEC.

### Histones reduce endothelial activated protein C generation

**Figure 4:**
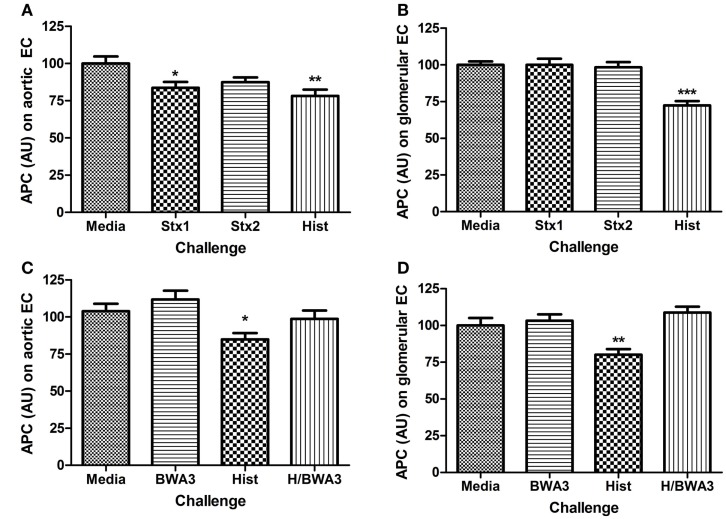
**Challenge with Stx or histones decreases activation of protein C**. The ability of human aortic endothelial cells **(A,C)** or renal endothelial cells **(B,D)** to activate protein C after 24 h challenge with Stx1, Stx2, or histones was determined on confluent monolayers with 10 nM human alpha-thrombin and 0.2 μM human protein C at 37°C. Activated protein C was quantified in cell supernatants with spectrozyme PCa substrate and converted to nM APC using a standard curve, then adjusted for cell number with Draq5 staining. Anti-histone antibody BWA3 (20 μg/mL) was added before challenge in some wells. Differences relative to media alone are shown as *p < 0.05, **p < 0.01, ***p < 0.001.

Functional changes resulting from alterations to surface expression of TM or EPCR ought to include changes to the rate of protein C activation. To determine if surface expression level changes of TM or EPCR correlated with functional changes that could be extrapolated to have significance in the development of HUS, the rate of protein C activation was determined on endothelial cells after challenge with Stx or histone DAMPs. Only Stx1 or histones had significant effects on the activation of protein C in aortic endothelial cells (Figure 4A). Histone challenge of HRGEC led to a significant decrease in the rate of protein C activation at 24 h post-challenge (Figure 4B; *p* < 0.001); however, neither Shiga toxin alone caused a significant difference in the amount of protein C activated. The suppression of protein C pathway function by histones was successfully prevented in the presence of anti-histone monoclonal antibody BWA3, which inhibits H4 and H2a (40), and protein C activation was restored on both aortic and glomerular endothelial cells in the presence of the blocking antibody during challenge with histones (Figures 4C,D).

### Histones but not Stx enhance thrombin-induced loss of endothelial barrier function

**Figure 5:**
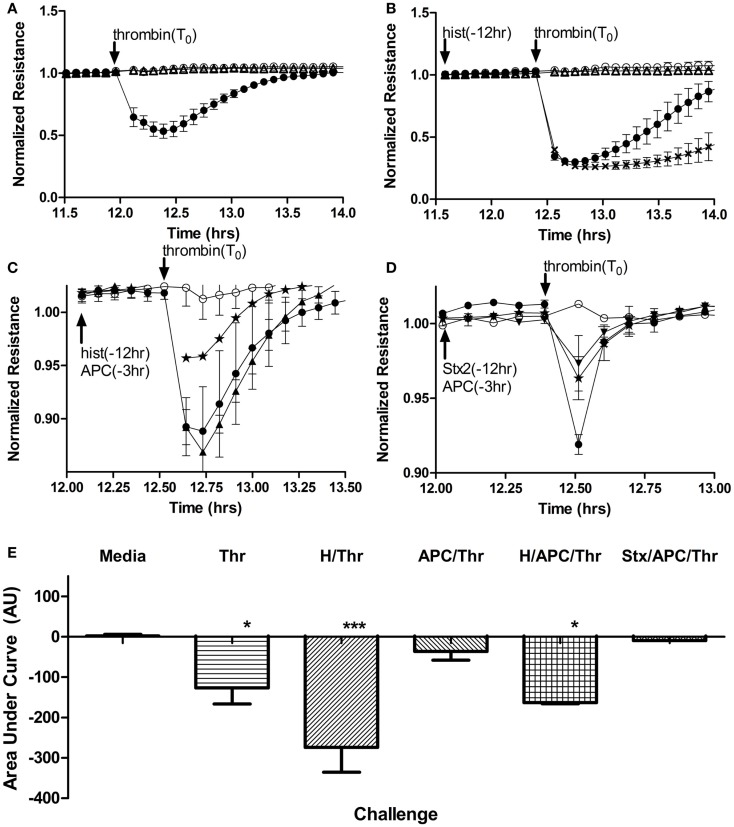
**Histones, but not Stx, contribute to increased endothelial cell permeability and block APC rescue**. Permeability of human aortic endothelial cells was determined by monitoring changes in electrical resistance across a monolayer as described in Section “Materials and Methods.” **(A)** Steady state resistance observed with media (○) decreases after addition of 1 U/mL (16.85 nM) human thrombin (●), reflecting increased permeability. Challenge with Stx2 (∆) lead to no change in permeability. **(B)** Increased permeability after thrombin (●) challenge is significantly prolonged if cells are pre-exposed to 50 μg/mL histones (**X**) for 12 h, but again, Stx2 causes no appreciable change in permeability (∆). **(C)** Compared to thrombin (2 nM) alone (●), APC pre-exposure (⋆) attenuates thrombin effects on permeability, but cannot rescue permeability of the cells if they have also been exposed to histones (▲). **(D)** Thrombin increased permeability (●) and APC rescued cells (⋆), but adding 100 ng/mL Stx2 (▼) did not change the protective effect of APC. **(E)** Permeability data were quantified as area under the curves; mean ± SD of three to four experiments each. Neither Stx1 nor Stx2 alone altered electrical resistance of the monolayers and so Stx2 is shown on graphs as representative of either Shiga toxin. *p < 0.05, ***p < 0.001.

Along with their roles in anticoagulation, both endothelium and APC have important roles in maintaining a selective barrier in the vasculature. Endothelial cell permeability changes in response to Stx and histones were examined by monitoring the resistance of a human aortic endothelial monolayer in a continuous fashion using an electrical cell impedance system. As expected, thrombin (1 U/mL final) stimulation resulted in a rapid decrease in resistance (Figure 5A), which correlates with increased permeability. However, in cells pre-treated for 12 h with histones (25 μg/mL) prior to thrombin stimulation, the thrombin-induced barrier function decrease was prolonged (Figure 5B). Recovery to pre-treatment levels following thrombin stimulation occurred after ~2 h; recovery from thrombin on histone-treated endothelium had not yet occurred by 18 h post-challenge (data not shown for presentation purposes). Pre-treatment with APC abrogated thrombin’s effect on monolayer permeability (Figure 5C), consistent with the known barrier protection properties of APC. APC was not able to rescue the loss of cell permeability due to thrombin when the cells were pre-treated with histones (Figure 5C), and treatment with histones alone did not increase permeability (data not shown). Stx2 did not change the duration of increased permeability of HAEC to thrombin, and APC was able to restore cells from thrombin-induced leak regardless of whether or not Stx2 was present (Figure 5D). Challenge with Stx1 did not alter these results. The area under the curves was calculated to quantify the relative effects (Figure 5E). Thus, under these conditions, histone DAMPs greatly exacerbate thrombin’s impact and prevent APC from being protective, though the toxins do not appreciably change endothelial barrier function.

### DAMPs are present in the plasma of Stx2-challenged mice

**Figure 6:**
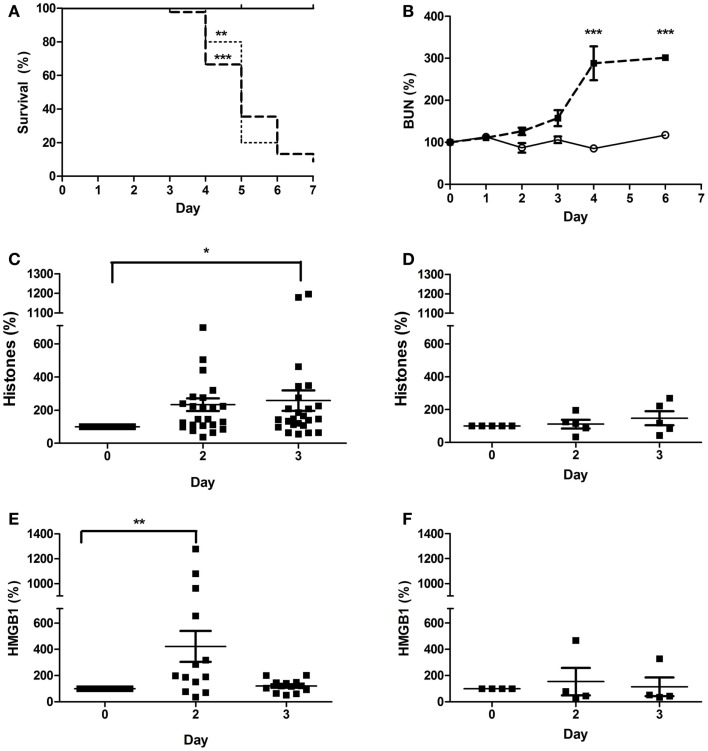
**Plasma DAMPs increase after Stx2 challenge in mice**. Mice were challenged i.p. with 1 ng/20 g body weight Stx2 [bold dashed line in **(A)**, black squares in **(B–F)**, *n* = 45] or saline [solid line in **(A)**, open circle in **(B)**, *n* = 15] on Day 0 and Day 3 and monitored for **(A)** survival and **(B)** plasma BUN. Plasma histones **(C,D)** and HMGB1 **(E,F)** were measured by ELISA. One group received a bolus dose of anti-histone BWA3 (400 μg i.p.) before challenge [**(D,F)**; *n* = 5]. Survival was not influenced by BWA3 [**(A)**, dotted; *n* = 5]. *p < 0.05, **p < 0.01, ***p < 0.001.

In order to determine whether Stx2 challenge produces DAMPs in mice, we used two different mouse models: a Stx2-toxemia model, and a Stx2-producing bacterial model. Mice challenged with Stx2 (1 ng/20 g body weight; i.p.) on day 0 and day 3 exhibited decreased survival (Figure 6A, dashed line) and kidney injury evidenced by increasing BUN relative to saline controls (Figure 6B; *p* < 0.001 on day 4). Plasma histone levels in Stx2 challenged mice were significantly elevated on day 3 post-challenge (258% ± 62% *n* = 24) when compared to day 0 (Figure 6C). Plasma levels of HMGB1, another DAMP associated with disease severity in sepsis models, were significantly elevated by 2 days after Stx2 challenge (421% ± 117%; *n* = 13) when compared to day 0 (Figure 6E). Pre-treatment with anti-histone BWA3 antibody prevented the significant rise in plasma histones (Figure 6D). Surprisingly, pre-treatment with anti-histone BWA3 antibody also abrogated the rise in HMGB1 (Figure 6F). Despite the reduction in circulating DAMPs by antibody, there was no alteration in survival outcome after lethal Stx2 challenge (Figure 6A, dotted). This result was not wholly unexpected, as the renal damage in mice is centered more in the tubular epithelium and collecting ducts, as opposed to the renal glomerular endothelium in humans, and the mice do not display thrombotic microangiopathy.

**Figure 7:**
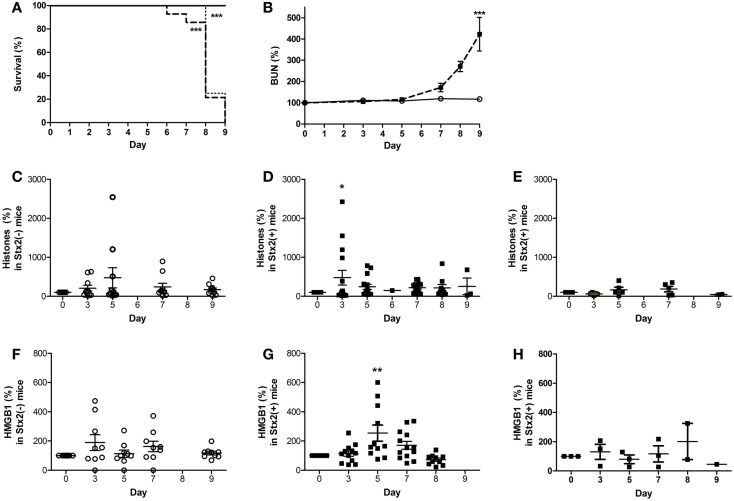
**Plasma DAMPs increase during intestinal infection with Stx2+ *Citrobacter rodentium***. Mice were gavaged with 1 × 10^9^ CFU Stx2+ *C. rodentium* [bold dashed line in **(A)**, black square in **(B,D,E,G,H)**, *n* = 15] or Stx2− strain *C. rodentium* [solid line in **(A)**, open circle in **(B,C,F)**, *n* = 10] on Day 0 and monitored for **(A)** survival and **(B)** plasma BUN. Plasma DAMPs were measured as histones or HMGB1 in Stx2− mice **(C,F)** and Stx2+ mice **(D,G)**. Baseline levels of both DAMPs were observed when anti-histone antibody BWA3 (400 μg; i.p.) was injected 10 min prior to challenge with Stx2+ bacteria [**(E,H)**; *n* = 5/group]. Pre-treatment with BWA3 did not affect survival after challenge with the Stx2+ strain [**(A)**, dotted line; *n* = 5]. *p < 0.05, **p < 0.01, ***p < 0.001.

During EHEC infection, Stx exposure occurs over days as the gut is colonized by EHEC that produce and release the Shiga toxins, so we also utilized a murine model in which mice are infected by oral gavage with *C. rodentium* strains that either do or do not express Stx2. The intestinal attaching/effacing lesions and contributions of Stx2 toward organ injury have been described (41), and this model has been shown to produce similar A/E lesions and thus be more relevant than a Stx2-alone model. Inoculating the mice by oral gavage with ~1 × 10^9^ CFU of *C. rodentium*-Stx2 resulted in decreased survival (Figure 7A; dashed line) compared to mice that received control *C. rodentium*, which does not express the toxin. As expected, kidney injury was toxin-dependent as evidenced by elevated plasma BUN by days 7–9 with the toxin-producing strain (Figure 7B; *p* < 0.001 on day 9). Mice challenged with non-Stx2 producing *C. rodentium* (open circles) did not develop changes in plasma levels of extracellular histones for the duration of the experiment when compared to day 0 (Figure 7C). In contrast, mice challenged with *C. rodentium*-Stx2 (black squares) developed significantly increased plasma levels of histones on day 3. This effect was significant when compared their own day 0 values and was also significant compared to plasma histone levels in non-Stx2 producing *C. rodentium* challenged mice (Figure 7D). Plasma HMGB1 did not increase significantly in mice challenged with non-Stx2 producing *C. rodentium* (Figure 7F), in contrast to elevated levels of HMGB1 by day 5 in *C. rodentium*-Stx2 mice (Figure 7G). BWA3 anti-histone antibody pre-treatment (dotted line) did not alter survival. Survival was still significantly decreased (*p* < 0.001) compared to animals challenged with *C. rodentium* not expressing Stx2, and there was no significant difference when compared with mice given Stx2-expressing *C. rodentium* and no antibody pre-treatment. The observed increase in plasma histone and HMGB1 levels were abolished by treatment with the anti-histone BWA3 antibody (Figures 7E,H). In conclusion, both murine models demonstrated that DAMPs were consistently elevated when Stx2 was present, and in both cases the rise occurred prior to rise in BUN, confirming that in the presence of Stx2, DAMPs are produced.

## Discussion

### Low relative expression of protective EPCR and TM on renal glomerular endothelial cells

Differential expression of EPCR and TM between large vessel endothelium and the microvasculature is known from immunohistochemistry studies, particularly for EPCR, which is expressed relatively more on large vessel endothelium (42). Here, we provide quantitative assessment of EPCR, TM, and PAR1 expression on cultured primary endothelial cells using on-cell western. Our results show that there are 3.2-fold lower and 7.4-fold lower expressions of TM and EPCR, respectively, on renal glomerular endothelial cells when compared with aortic endothelial cells in the same experiment. APC is protective in acute kidney injury murine models (43), and the organ system most vulnerable to the sequelae of low EPCR expression is the kidney, which demonstrates enhanced albuminuria and profound renal hemorrhage after LPS challenge (44). The kidneys are already a toxin target due to glomerular Gb_3_ expression, and the current data suggest that further reduction due to toxin and DAMP exposure of already low TM and EPCR expression may provide an environment highly susceptible to thrombus production.

### *In vitro* loss of EPCR and TM surface expression on endothelial cells due to Stx and DAMPs

D + HUS is characterized by thrombotic microangiopathy, or a forming of microthombi in the small vasculature of the glomerulus, suggesting loss of endothelial anti-coagulant capacity. The receptors and enzymes that make up the protein C pathway of endothelial cells are critical contributors to intact anti-coagulant function of the endothelium and loss of these anti-coagulant and cytoprotective molecules could have significant pro-thrombotic consequences. Surface expression levels of protein C pathway molecules are changed in Shiga toxin- or DAMPs-challenged human endothelial cells, as demonstrated here *in vitro*. Shiga toxin and/or DAMP challenge of human endothelial cells resulted in a shift toward a pro-coagulant environment, and this has functional consequences with respect to the generation of APC, an enzyme critical in controlling thrombus formation. We made several significant observations, including reduced APC generation, enhancement of endothelial barrier permeability, and reduction of the ability of APC to protect barrier function in the context of Stx or histone DAMP challenge. These data provide insights into the mechanism of Stx-induced endothelial dysfunction in D + HUS and strongly suggest a potential role for tissue damage induced DAMPs in this process.

Thrombin bound to TM preferentially cleaves and activates protein C instead of fibrinogen (45), creating APC and ultimately reducing fibrin clot formation. EPCR makes the thrombin/TM cleavage of protein C even more favorable. EPCR also plays a crucial role in endothelial function because APC/EPCR complex activation of PAR1 leads to a cytoprotective signaling cascade (19, 46, 47). We previously demonstrated in a non-human primate model of HUS that Shiga toxins alone can cause HUS and acute renal failure with fibrin deposition and swelling and detachment of glomerular endothelial cells (48, 49). Cellular mechanisms are difficult to study in non-human primates and mouse models do not develop the coagulopathy of HUS, so the current study with human cells extends the *in vivo* observations by demonstrating *in vitro* loss of EPCR and TM surface expression on endothelial cells due to Stx and DAMPs, particularly histones, and reduced capacity to generate APC. A pro-thrombotic environment is further created by DAMP-induced increased expression of PAR-1, the pro-thrombotic thrombin receptor (50). Both histones and Shiga toxins reduced EPCR expression, and Shiga toxins also lowered TM on HRGEC, with the end result that both Stx1 and histones affected the amount of APC generated by HRGEC in HAEC, with histones alone affecting APC production in HRGEC.

### Loss of *in vitro* endothelial barrier function due to histones but not Stx

D + HUS patients can develop edema, and in our system we observed loss of *in vitro* endothelial barrier function primarily due to histones, rather than through any effect of Stx1 or Stx2 on endothelial cells. Histones increased PAR-1 and lowered EPCR expression and pre-treatment of human aortic endothelial monolayers with histones significantly delayed the ability of the endothelial barrier to recover to baseline after thrombin challenge. Histones also significantly inhibited the barrier-protective effects of APC. It is possible that histones might compete as a substrate for APC (32), but we did not find any effect of histones on APC chromogenic activity even at histone concentrations up to fourfold higher than those used here (data not shown). PAR1 can function in both barrier protective and disruptive capacities, depending on which protease cleaves it and whether EPCR is occupied by a ligand. Shiga toxins and DAMPs both decrease the surface ratio of EPCR:PAR1 by lowering EPCR; however, only histones also raise PAR1, which could account for the differential effects of histones vs. Shiga toxins with respect to APC inhibition. We hypothesize that the combination of decreased EPCR along with increased PAR1 creates more opportunity for thrombin signaling while dampening the potential protective effects of EPCR occupied with APC, helping to explain the prolongation of the permeability effects of thrombin despite its use.

### DAMPs are produced by the action of Shiga toxins in mice

The purpose of our mouse models was to demonstrate that DAMPs are produced by the action of Shiga toxins. The finding of significantly elevated DAMPs is to the best of our knowledge a novel finding in mouse models of Shiga toxin challenge and is consistent with prior studies in our non-human primate Stx-HUS models (51). The finding that levels of plasma HMGB1 in both mouse models decreased after an initial significant rise instead of continuing to rise with disease severity could be due to the limitations of current mouse models for this disease, because the distribution of Gb_3_ receptors in the mouse differs from that in humans or non-human primates (4, 5, 49). Unlike in human kidneys, Shiga toxins target renal tubule epithelial cells in mice (6) giving rise to the possibility that HMGB1 was excreted as urination becomes increasingly excessive in the mouse models due to tubular injury. In contrast, humans and baboons express Gb_3_ on endothelial cells, particularly in the glomeruli, and disease severity is accompanied by progressive anuria so that the baboon Stx challenge models have and increase in certain plasma DAMPs such as mitochondrial DNA over time (35). However, in contrast, we have previously shown that HMGB1 in non-human primate models also initially rises before dropping (51), so the waxing or waning pattern of different DAMPs in the serum with Shiga toxin challenge likely relies on mechanisms that remain to be understood and fall outside the scope of this discussion.

In our experiments, we introduced the anti-histone antibody BWA3, which targets H4 and H2a, and is derived from an autoimmune mouse model (40). A bolus dose of 400 μg BWA3 prior to Stx2 or *C. rodentium*-Stx2 significantly decreased plasma levels of histones and HMGB1, although in neither case was the outcome altered for the model. These results suggest that these DAMPs may function more as markers of cell injury in these murine models rather than driving pathology as they might in other models; however, it was not our intention to investigate endothelial injury in our murine models, for reasons, which have been stated already. For this purpose, we turned to *in vitro* models of endothelial cell injury. Our human endothelial cell experiments show a significant impact by DAMPs with respect to APC generation and endothelial dysfunction, but this is not possible to replicate in the mice, as these models exhibit Stx-induced tubular epithelial injury (5), with no evidence of glomerular endothelial damage, thrombotic microangiopathy, or thrombocytopenia of HUS as stated before. These are limitations of all mouse models using endotoxin-free Stx challenge. Other investigators have shown that in mice challenged with LPS (a model of endothelial cell injury) BWA3 reduced circulating histones and improved survival (32). The current experiments with human endothelial cells raise the possibility that BWA3 might have more of an effect on outcomes in models of kidney injury driven by endothelial rather than tubular damage, such as a non-human primate model that more closely mimics human pathology (36, 37, 49); however, there are clear limitations to doing the experiments described in this paper in non-human primates, and so we chose to progress as described.

Elevated HMGB1 and histones have been shown experimentally to be involved in SIRS development by the fact that inhibition of either is beneficial in animal models of LPS-induced inflammation (32, 33). Currently, development of HUS in patients during EHEC infection is largely attributed to the bacterially produced Shiga toxin effects on endothelium or platelets (52), but the current data suggest that the host response to injury contributes as well. During active infection, Shiga toxins rapidly become undetectable in the plasma days before development of HUS (39). During this time, cells internalize the toxins, the intestinal bacteria damage the intestinal epithelium, and both processes may lead to release of DAMPs. The observations made here cannot link DAMP activities directly to susceptibility of patients to develop HUS, but they do provide insight into a possible molecular basis for development of increased vascular permeability and a pro-thrombotic environment in the context of Stx2-induced tissue damage. We showed this by demonstrating that DAMPs are produced by the Shiga toxins, and that histone DAMPs cause demonstrable endothelial dysfunction in two types of human endothelial cells.

### Limitations of the current study and future directions

While we have made several important observation in the experiments described here promoting a possible role for Stx2-induced DAMPs in the development of endothelial dysfunction underlying HUS, there are still important questions that remain unanswered. We did not identify cellular mechanisms linking Shiga toxins and extracellular histones to the regulation of EPCR, TM, and PAR1 endothelial expression, as we were more interested in the end consequence of reduced protein C activation; however, this mechanism could also be a source of potential therapeutic targets and warrants investigation. Matrix metalloproteinases and MAPK pathways may contribute to EPCR shedding (53, 54), though how these may be impacted by Stx or DAMPs in this context was not addressed. Histones act through TLR4 (55), which might induce PAR1 through a similar mechanism, though we do not address this here. Finally, Stx1 and Stx2 interact with human neutrophils via TLR4 (56), but how that might impact histone or other DAMP activities is not known. Pilot experiments using TAK-1, JNK, and IRAK1/4 inhibitors demonstrated no significant prevention of Shiga toxin effects on EPCR, PAR1, or TM (data not shown). The largest limitation of our studies was the lack of a mouse model demonstrating full-spectrum HUS, which to date has only been reproducibly done by adding LPS to the Stx challenge (6, 57, 58). Ideally, we would test our hypothesis *in vivo* using animal models that demonstrate kidney injury driven by endothelial dysfunction, and then we could show whether DAMPs are playing a significant role by using therapies such as BWA3 and quantifiable outcomes such as biomarkers and survival. Additionally, quantifying the presence of DAMPs in the blood of patients with HUS would be a good next step prior to experiments with full-spectrum HUS animal models. Previous work has already amply demonstrated the pathology and tissue damage (25, 59), and quantifying the DAMPs that are almost certainly present would provide the evidence needed to investigate whether they function as markers of disease impact or drivers of disease. The data here lay groundwork, giving evidence that this could be a fruitful path for future investigations.

## Conflict of Interest Statement

The authors declare that the research was conducted in the absence of any commercial or financial relationships that could be construed as a potential conflict of interest.
